# Examining the dimensionality, reliability, and invariance of the Chinese version of the Resilience Scale-14: A multicenter cross-sectional survey in Chinese junior nurses

**DOI:** 10.3389/fpsyt.2022.964151

**Published:** 2022-11-03

**Authors:** Weidong Zhao, Ting Shu, Yue Ma, Xuemei Wei, Cairong Zhu, Liping Peng, Lihong Zhao, Qin Zhang

**Affiliations:** ^1^West China Hospital, Sichuan University, Chengdu, China; ^2^West China School of Public Health and West China Fourth Hospital, Sichuan University, Chengdu, China; ^3^Department of Integrated Traditional Chinese and Western Medicine, West China Hospital, Sichuan University, Chengdu, China; ^4^Affiliated Hospital of North Sichuan Medical College, Nanchong, China; ^5^Department of Radiology, West China Hospital, Sichuan University, Chengdu, China

**Keywords:** 14-item Resilience Scale (RS-14), Chinese nurses, confirmatory factor analysis (CFA), measurement invariance, cross-sectional

## Abstract

**Background:**

This study aimed to present the psychometric properties (dimensionality, reliability, and invariance) of the Chinese 14-Item Resilience Scale (RS-14) within Chinese register nurses (RNs) with less than 3 years work experiences. And we aimed to compare the fit of a unidimensional model and a bifactor model.

**Materials and methods:**

This multicenter cross-sectional survey was conducted from August to September in 2019. A total of 7,231 registered nurses from 90 hospitals were recruited. Data was anonymously obtained through online questionnaires. Both reliability and validity of Chinese RS-14 were assessed. The confirmatory factor analyses (CFA) were used to compare the fit of two different factor structures of the RS-14 (unidimensional model vs. a bifactor model). Moreover, multigroup CFA (MGCFA) were applied to evaluate the measurement invariance (MI) across sociodemographic parameters (gender, educational level, marital status, and et al.).

**Design:**

Cross-sectional quantitative analyses.

**Results:**

Our study confirmed that the bifactor model presented the best fit within Chinese nurses (CFI = 0.924, TLI = 0.909, RMSEA = 0.095, SRMR = 0.043), and found strong factorial invariance across gender, marital status, and status of receiving standardized training. The reliability of RS-14 was high with a Cronbach’s alpha coefficient of 0.939. Moreover, RS-14 was positively correlated with the social support and was negatively correlated with workplace bullying.

**Conclusion:**

This is the first study to explore the latent factor structure for the RS-14 among Chinese RNs and evaluated MI across a series of sociodemographic variables. Based on our findings, the Chinese version RS-14 is both valid and reliable.

## Introduction

In the initial period of clinical nursing practice, register nurses (RNs) were exposed to several stressors, including heavy workloads, inadequate communication skills, high exposure to unprofessional workplace, expertise-beyonding responsibilities, limited support, as well as disempowerment (1–3). These stressors were associated with chronic physical problems, such as low back pain and musculoskeletal symptoms (4, 5). Moreover, stress also led to psychological problems, such as distress, burnout, depression, and anxiety, which deteriorated the patients’ quality of life. Futhermore, staff exhaustion and intention to leave bring harm to hospital efficiency (6). Previous studies showed that some of RNs showed remarkable resilience, though exposed to stressful clinical environments, which assisted them coping with workplace stress better, and performing better in work (7). Resilience was the capacity to respond to stress in a healthy way such that goals are achieved at minimal psychological and physical cost, has been identified as a key to enhancing quality of care, and sustainability of the health care workforce. Accumulating evidence indicates that resilience is an effective protective factor (8, 9). It could help nurses transform adversities into positive growth experiences, which contributes to nurses’ professional development and better mental and physical health.

The Resilience Scale (RS) was initially developed by Wagnild and Young to determine the degree of individual psychological resilience (10). It was a 7-Likert point scale ranging from 1 (disagree) to 7 (agree) and comprised 25 items, therefore, the original RS came to be designated as RS-25. Although the RS-25 is very popular, the element structure of the RS-25 is still controversial (11). The 14-Item Resilience Scale (RS-14) has been developed as a newer and shorter version of the resilience scale by Wagnild, and has been applied in Western countries (12). The RS-14 is a brief, quick-to-complete, user-friendly measurement tool, and the psychometric characteristics of the RS-14 have been suggested to be comparable to those of the RS-25 (12). RS-14 has shown excellent reliability and validity in various populations, including adolescents, young adults, nursing students, elderly individuals, and community residents (13–18). However, the factor structure of the RS-14 has had unclear results in previous studies: two-factor structure appeared more appropriate for the whole English, Japanese, and Chinese populations (14, 16, 18, 19), unidimensional structure of RS-14 was found to be more appropriate in Chinese community residents and Hong Kong Chinese adolescents (15, 17), while a it was found that the structures of three factor was suitable for Italian population (13). Social and cultural factors are highly relevant to how different groups are defined. Studies showed a variety of reliability and validity of RS-14 around the world (20). RS-14 is widely used in China to screen patients with tumors, cancer and other major diseases. No study has so far adequately explored the psychometric attributes of the RS-14 among Chinese RNs populations, which may manifest different resilience levels.

Moreover, prior studies revealed distinctions in the resilience levels of nurse populations with different sociodemographic variables, such as gender, marital status, educational level, and standardized training in reported use of the RS-14 based on ANOVA test or student *t*-test (9, 21). However, the traditional ANOVA test was not appropriate since measurement invariance was unclear (22). Measurement invariance (MI) was essential in repetitive comparisons that concerning other variables (i.e., time, metric, et al.) and could be estimated in four ways: configural invariance, metric invariance, scalar invariance, and strict invariance (22, 23). Only one study using the RS-14 assessed resilience levels between men and women as well as between races or ethnicities (21). Sociodemographic factors associated with nurse resilience were investigated, whereas, mixed evidence restricted its application (9). No study reported the MI of the RS-14 of Chinese nurses.

The primary purpose of our study was to evaluate the dimensionality and reliability of the RS-14 by analyzing two theoretically plausible models (factor 1 and factor 2 structure models) in Chinese nurses with less than 3 years work experiences since previous studies suggested factor 1 and factor 2 structure models were fit in Asian population. Our secondary purpose was to examine the configural, metric, scalar and strict invariance of the RS-14 by gender, educational level, marital status, and standardized training in our sample and to determine which model was more suitable for the population. Moreover, the variables of the Social Support Rating Scale-utilization of social support (SSRS-USS) and workplace bullying were considered concurrent validity indicators of the RS-14, and we would like to explore the correlations between RS-14 and SSRS-USS.

## Materials and methods

### Participants

From August to September 2019, Registered nurses (RNs) who had less than 3 years work experiences from 90 hospitals were recruited. Participants were excluded if diagnosed with psychiatric diseases or refusal to give consent. According to the literature, sample size needed to be 10 times more than the number of items; thus, the study tried to obtain the maximum sample size.

### Design

This is a cross-sectional and self-reported study based on quantitative data. Sampling and data collection have been carried out completely online. Anonymous online questionnaire was used to collected data.

### Ethical considerations

Our study was approved by Ethics Committee of West China Hospital of Sichuan University (No. 2019-435). Participants received the invitation for the online survey via their work email or mobile phone. Informed consent was obtained at the beginning of the survey.

### Data analysis

SPSS 23.0 was used for data analysis Kolmogorov–Smirnov test of normality (*D* test) was performed to determine the normality of the total RS-14 score. We calculated mean scores, standard deviations (SDs), interquartile ranges (IQRs), and range statistics for the RS-14). AMOS 24.0 was applied for component factor analysis (CFA). The *P* value < 0.05 was considered statistically significant.

CFAs were conducted in the RS-14; these analyses were conducted to examine the fit of the following two models of the RS-14 in a sample of Chinese RNs: a one-factor structure model (based on research conducted by the developer of the original English scale) and a two-factor structure model (based on research conducted by the developer of the Chinese version). A maximum likelihood estimation was performed on both models using AMOS. Based on the recommendations of previous studies (24, 25), a good fit was determined by examining the following criteria: χ^2^ (df), Tucker-Lewis index (TLI), comparative fit index (CFI), standardized root mean square residual (SRMR), root mean square error of approximation including 90% confidence interval (RMSEA and 90% CI), Akaike information criterion (AIC), and the Bayesian information criterion (BIC). Furthermore, the latter indexes were used together with χ^2^ values because sample size makes the value of χ^2^ inflated, and thus, it is routine for large-N solutions to be rejected on the basis of χ^2^ (20). SRMR indicates a good fit at low values (close to 0.08 or below), RMSEA also indicates a good fit at low values (close to 0.06 or below, 0.08–0.10 mediocre fit), while TLI and CFI indicate a good fit at high values (close to 0.95 or greater, 0.90–0.95 acceptable model fit) (20). A CFA-based method was used to evaluate measurement invariance across samples to ensure that the observed group differences in the RS-14 scores were not affected by measurement artifacts (26). Multiple group confirmatory factor analysis (MGCFA) was then examined by demographic variables (gender, academic degree, marital status, and status of receiving standardized training) with the better-fitting model. Considering the substantial differences in sample size among demographic variables, the subgroup with a larger sample was selected at random that matched groups with the small samples (21, 27). Stepwise procedures were employed to test measurement invariance as follows the parameters measured: (I) the CFA model was separately tested in each group; (II) a simultaneous test of the equal form (configural invariance); (III) the equality of factor loadings (metric invariance); (IV) the equality of indicator intercepts (scalar invariance); and (V) the equality of indicator residual variances (optional, test of strict invariance). The degree of invariance was assessed by ΔCFI, where the null hypothesis of invariance should not be rejected as long as a value is less than or equal to –0.01 (28).

### Validity and reliability/rigor

To demonstrate differences within samples, we estimated the latent mean. Group comparisons of the latent mean are meaningful only if the factor loadings and indicator intercepts have been found to be invariant. The mean structure component of the multiple-group solution is under identified in the absence of additional restrictions, so we fixed the latent mean to zero in one group and freely estimated the remaining groups. This means that these parameter estimates represent the deviation from the potential mean of the reference group (20). The modified Bonferroni procedure to control error rates was used to compare the latent mean of multiple groups (29, 30). Third, in calculating Cronbach’s alpha coefficient, we checked the consistency of the indicators in the questionnaire. The split-half reliability of the scale was calculated using Spearman-Brown’s coefficient. Spearman’s correlation analysis between the RS-14 and SSRS-USS scores as well as workplace bullying scores was used to examine concurrent validity.

### Instruments

The simplified Chinese version of the Resilience Scale-14 (17) is a 14-item instrument with a 2-factor structure that assesses personal competence (PC) and acceptance of self and life, ranging from a score of 1 (strongly disagree) to 7 (strongly agree) with an Cronbach’s alpha (α) of 0.93. The Likert scale was changed from 7-points to 5-points to reduce the response time (31).

Previous studies showed that resilience measured by the RS or RS-14 was positively correlated with social support (18). We investigate the level of social support and workplace bullying by the Utilization of Social Support (USS). USS is a dimension of Xiao’s SSRS (Social Support Rating Scale), consisting of three items rated on a 4-point Likert scale, which measures the behavior patterns adopted by individuals when seeking social support (32). SSRS was measured by the total scores of responses to six statements from a previous study (24) with 0 indicating “never” and five indicating “frequent.” The internal consistency of the USS was 0.738 and for workplace bullying was 0.860.

## Results

### Participants

The subjects comprised 516 (7.1%) junior male nurses and 6,715 (92.9%) junior female nurses aged 18–30 years (median = 23.00, IQR = 22.00–24.00). Female nurses and male nurses did not differ significantly in age (mean = 23.10 vs. mean = 23.02; *P* = 0.280). The subjects were relatively highly educated, with 179 (2.5%) technical secondary school degrees, 5,258 (72.7%) associate degrees, and 1,794 (24.8%) bachelor’s degrees or higher. Among the subjects, 527 (7.3%) did not receive standardized training, 3,091 (42.7%) had completed standardized training, and 3,613 (50.0%) were participating in standardized training. After excluding data from 268 participants who completed multiple questionnaires or did not comply with the requirements, an analysis of data from 7,231 junior nurses was conducted.

### Confirmatory factor analysis

CFA was conducted to determine how well the RS-14 data from the Chinese junior nurse population fit the previously presented factor solutions. Table 1 shows the results of the CFA. All factor loadings in both models were found to be higher than 0.40. The range of factor loadings in the 1-factor model was from 0.482 to 0.857, while factor 1 loadings ranged from 0.627 to 0.853, and factor 2 loadings ranged from 0.476 to 0.846 in the 2-factor model. Factor 1 includes ten items that indicate self-control and personal qualities, so it is considered the PC factor. Factor 2 includes four items involved in individuals’ positive awareness and lives, defining it as the PP factor.

**TABLE 1:** RS-14 items, standardized factor loadings, and model fit indexes (*n* = 7,231).

Item No.	Item	1-factor model	2-factor model
RS-14-01	I usually manage one way or another	0.682	0.693 (PC)
RS-14-02	I feel proud that I have accomplished things in life	0.618	0.627 (PC)
RS-14-03	I usually take things in stride	0.711	0.727 (PC)
RS-14-04	I am friends with myself	0.482	0.476 (PP)
RS-14-05	I feel that I can handle many things at a time	0.688	0.703 (PC)
RS-14-06	I am determined	0.776	0.784 (PC)
RS-14-07	I can get through difficult times because I have experienced difficulty before	0.741	0.750 (PC)
RS-14-08	I have self-discipline	0.673	0.679 (PC)
RS-14-09	I keep interested in things	0.757	0.826 (PP)
RS-14-10	I can usually find something to laugh about	0.777	0.846 (PP)
RS-14-11	My belief in myself gets me through hard times	0.823	0.819 (PC)
RS-14-12	In an emergency, I am someone people can generally rely on	0.759	0.762 (PC)
RS-14-13	My life has meaning	0.807	0.829 (PP)
RS-14-14	When I am in a difficult situation, I can usually find my way out of it	0.857	0.853 (PC)
**Model fit indexes**			
χ^2^ [df]		6,322.229 [77]	5,043.763 [76]
RMSEA (90% CI)		0.106 [0.104, 0.108]	0.095 [0.093, 0.097]
SRMR		0.046	0.043
TLI		0.888	0.909
CFI		0.905	0.924
AIC		6,378.229	5,101.763
BIC		6,571.041	5,301.461

PC, personal competence factor; PP, positive perception factor; RMSEA, root mean square error of approximation; 90% CI, 90% confidence interval for RMSEA; SRMR, standardized root mean square residual; TLI, Tucker-Lewis index; CFI, comparative fit index; AIC, Akaike information criterion; BIC, Bayesian information criterion.

RMSEA (0.106) and TLI (0.888) were rejected, whereas SRMR (0.046) and CFI (0.905) were acceptable in the one-factor model. The fit indexes of CFI (0.924), TLI (0.909), RMSEA (0.095), and SRMR (0.043) for the two-factor model were found to be satisfactory. The AIC and BIC values of the two-factor model were likewise lower. The two-factor model of the RS-14 showed a better fit for data among junior nurses than the one-factor model.

### Tests of measurement invariance

The MGCFA was then examined by sociodemographic features (gender, academic degree, marital status, and status of receiving standardized training) with the 2-factor model. Table 2 displays all multigroup models, along with their fit indexes and sample sizes. Gender was found to be invariant across all models. Table 2 displays the results of the two-factor model. These findings indicate that both males and females well received the model. Then we ran MGCFA, and the equal form model fit the data perfectly (RMSEA = 0.067, SRMR = 0.043, TLI = 0.912, CFI = 0.927). The loadings for configural invariance were higher than 0.40 for both the male (all loadings 0.51–0.88) and female (all loadings 0.50–0.87) participants (not shown in the table). The following tests of measurement invariance were performed based on this solution. All other steps remained invariant, with only minor CFI reductions. (ΔCFI < 0.01). The RS-14 proved design invariance by gender in this investigation, suggesting that the model structure, factor loadings, indicator intercepts, and measurement residuals were all the same for men and women.

**TABLE 2:** Tests of measurement invariance of the RS-14 in a population with different demographic characteristics.

CFA models	χ^2^	df	RMSEA (90% CI)	SRMR	TLI	CFI	Δ CFI
Gender							
**Single-group solutions**							
Male (*n* = 516)	418.260	76	0.094 [0.085, 0.102]	0.043	0.915	0.929	
Female (*n* = 516)	441.941	76	0.097 [0.088, 0.106]	0.044	0.910	0.925	
**Measurement invariance**							
Configural invariance	860.201	152	0.067 [0.063, 0.072]	0.043	0.912	0.927	
Metric invariance	868.494	164	0.065 [0.060, 0.069]	0.043	0.919	0.927	0.000
Scalar invariance	923.173	178	0.064 [0.060, 0.068]	0.044	0.921	0.923	–0.004
Strict invariance	945.091	192	0.062 [0.058, 0.066]	0.045	0.926	0.922	–0.001
**Marital status**							
Single-group solutions							
Single (*n* = 579)	539.036	76	0.103 [0.095, 0.111]	0.052	0.888	0.907	
Married (*n* = 579)	584.021	76	0.108 [0.099, 0.116]	0.050	0.899	0.916	
**Measurement invariance**							
Configural invariance	1,123.057	152	0.074 [0.070, 0.078]	0.052	0.894	0.912	
Metric invariance	1,129.859	164	0.071 [0.067, 0.075]	0.051	0.903	0.912	0.000
Scalar invariance	1,174.766	178	0.070 [0.066, 0.073]	0.051	0.907	0.909	–0.003
Strict invariance	1,210.832	192	0.068 [0.064, 0.071]	0.052	0.912	0.907	–0.002
**Status of receiving standardized training**							
**Single-group solutions**							
Without standardized training (*n* = 527)	476.103	76	0.100 [0.092, 0.109]	0.049	0.904	0.920	
Completed standardized training (*n* = 527)	515.341	76	0.105 [0.096, 0.114]	0.050	0.893	0.911	
In standardized training (*n* = 527)	525.236	76	0.106 [0.098, 0.115]	0.055	0.886	0.905	
**Measurement invariance**							
Configural invariance	1,516.679	228	0.060 [0.057, 0.063]	0.049	0.894	0.912	
Metric invariance	1,562.337	252	0.057 [0.055, 0.060]	0.054	0.903	0.910	–0.002
Scalar invariance	1,611.174	280	0.057 [0.054, 0.060]	0.054	0.911	0.909	–0.001
Strict invariance	1,692.163	308	0.055 [0.052, 0.058]	0.055	0.916	0.905	–0.004

χ^2^, model χ^2^; CFA, confirmatory factor analysis; RMSEA, root mean square error of approximation; 90% CI, 90% confidence interval for RMSEA; SRMR, standardized root mean square residual; TLI, Tucker-Lewis index; ΔCFI, CFI difference between 2 models.

For marital status, academic degree, and status of receiving standardized training analyses of the models, a pattern evolved in which the SRMR was within the expected range, the RMSEA was average or slightly above mediocre, and the TLI/CFI was approximately 0.90 or above. As recommended in the data analysis section, we investigated the combined structure of the nested models, and even if certain fit factors were not optimal, we analyzed the combined structure.

For marital status analyses, loadings for configural invariance were higher than 0.40 for both the single (all loadings 0.45–0.84) and married (all loadings 0.51–0.88) participants (not shown in the table). Further model fit was invariant until equal measurement residuals were examined. The invariance of each step was supported by a small drop in CFI (ΔCFI < 0.01).

For academic degrees, loadings for configural invariance showed a different result. The loadings of item 4 (I am friends with myself) in both the technical secondary school and associate degree groups were small, at 0.24 and 0.28, respectively. The model may have an item that is inappropriate for different academic degree groups. This result indicates that the configural invariance of the 2-factor model between the academic degree groups was rejected. That is, the measurement invariance was not satisfied.

For the status of receiving standardized training, the loadings for configural invariance were higher than 0.40 for all groups; those for the group without standardized training ranged from 0.49 to 0.88, those for the group that completed standardized training ranged from 0.43 to 0.84, and those for the group that participated in standardized training ranged from 0.44 to 0.87 (not shown in the table). Except for a few minor variations in CFI, the other phases of the study were consistent (ΔCFI < 0.01), which indicates that whether the data were determined by standardized training or not, the RS-14 exhibited measurement invariance with the same model structure, factor loadings, structural covariances, and measurement residuals.

### Group comparisons of latent mean

Next, latent mean analysis was conducted for gender, marital status, and status of receiving standardized training. The equal factor loadings and measurement intercept model demonstrated an excellent match to the data by gender (RMSEA = 0.063, SRMR = 0.043, TLI = 0.923, CFI = 0.925), marital status groups (RMSEA = 0.070, SRMR = 0.051, TLI = 0.907, CFI = 0.910), and status of receiving standardized training (RMSEA = 0.055, SRMR = 0.054, TLI = 0.910, CFI = 0.909), so we could estimate the latent mean and test the difference between subgroups. The female group’s latent mean of factor PC = –0.190 (*Z* = –4.397, *P* < 0.001) and PP = –0.114 (*Z* = –2.500, *P* = 0.012), which means the male participants showed a higher latent mean than the female participants on both latent factors of resilience. The married group’s latent mean for the PC factor = 0.084 (*Z* = 2.101, *P* = 0.036) and that for the PP factor = 0.065 (*Z* = 1.533, *P* = 0.125), indicating that, on average, the married group scored 0.084 units higher than the single group on the PC latent dimension based on the metric of the marker indicator. The latent mean PP was not significantly different from that of the single participants. For the status of receiving standardized training, we fixed the latent mean of the group without standardized training to 0 to estimate the potential mean of the other 2 groups. The latent mean of the PC factor for the group that completed standardized training was –0.073 (*Z* = –1.703, *P* = 0.088), and the latent mean of the PP factor was –0.035 (*Z* = –0.774, *P* = 0.439). The latent mean of the PC factor for the group that was receiving standardized training was –0.065 (*Z* = –1.593, *P* = 0.111), and the latent mean of the PP factor was 0.014 (*Z* = 0.319, *P* = 0.750). The difference in the latent means between the group that completed standardized training and the group that was receiving standardized training was 0.007 (*Z* = 0.179, *P* = 0.858) on the PC dimension and 0.049 (*Z* = 1.105, *P* = 0.269) on the PP dimension. The *P* values of all comparisons for differences in the latent means across the PP and PC dimensions were not significant.

### Reliability

A reliability test was conducted for the scale, and the results are presented in this section. Cronbach’s alpha for the RS-14 was 0.939. With the variables eliminated from the analysis, the Cronbach coefficients are shown in Table 3, and the alpha coefficients have not changed much. The range for interitem correlation (not shown in the table) was 0.481 (minimum 0.275 and maximum 0.757). The range for the corrected item-total correlation was 0.348 (minimum 0.470 and maximum 0.818). In the RS-14, a total of 14 items were classified into two sets (set 1: RS-14-1 to RS14-7, set 2: RS-14-8 to RS14-14). The correlation coefficient between the 2 sets was 0.813, and the split-half reliability was 0.897. With the reliability coefficients established for the scale, the RS-14 can thus be judged as a reliable scale for measuring resilience among junior nurses in China.

**TABLE 3:** Internal consistency-Cronbach’s coefficient of the RS-14 (*n* = 7,231).

Item	Item total correlation	If item deleted^a^
RS-14-01	0.673	0.935
RS-14-02	0.607	0.937
RS-14-03	0.707	0.934
RS-14-04	0.470	0.941
RS-14-05	0.684	0.935
RS-14-06	0.759	0.932
RS-14-07	0.717	0.934
RS-14-08	0.652	0.935
RS-14-09	0.723	0.933
RS-14-10	0.740	0.933
RS-14-11	0.785	0.932
RS-14-12	0.728	0.933
RS-14-13	0.767	0.932
RS-14-14	0.818	0.931

^a^Alpha value if item is deleted.

### Descriptive results of the RS-14

The RS-14 total score varied from 14 to 70 (median = 49.00, IQR = 43.00–55.00), and the total score was not normally distributed (*D* = 0.058, *P* < 0.001). The descriptive statistics for the total score, the PC factor, and PP factor are presented in Table 4.

**TABLE 4:** Descriptive statistics and test results for normality of RS-14 (*n* = 7,231).

Dimension	Mean	SD	Median	IQR	Range	Skewness	Kurtosis	*D*
RS-14 total score	49.49	8.32	49	43.00–55.00	14–70	0.074	0.367	0.058***
PC factor	34.84	6.09	35	30.00–39.00	10–50	0.091	0.368	0.062***
PP factor	14.65	2.49	15	13.00–16.00	4–20	–0.071	0.190	0.102***

SD, standard deviation; IQR, interquartile range; *D*, statistics of Kolmogorov–Smirnov normality test; ***P < 0.001.

### Validity support

The SSRS-USS scores of the participants ranged from 3 to 12 (median = 8.00, IQR = 7.00–10.00). As expected, the RS-14 and measures of social support utilization had a favorable connection (*r*_*s*_ = 0.378, *P* < 0.001), while the scores for workplace bullying ranged from 6 to 30 (median = 12.00, IQR = 9.00–16.00) and were negatively correlated with the RS-14 (*r*_*s*_ = –0.246, *P* < 0.001).

## Discussion

Our study measured the reliability and validity of the RS-14, confirmed its factor structure, analyzed measurement invariance, and compared resilience across sociodemographic parameters to establish its application in Chinese junior nurses. The RS-14 Chinese version preserved all 14 components, but a response scale with 5 points instead of 7 points was used. The study’s primary result was that the Chinese version of the RS-14 scored on a 5-point Likert scale had good reliability and validity among the junior nurse population. The results support the 2-factor structure obtained in previous studies in the Chinese population (17) and show the measurement invariance of resilience in various gender, marital status, and standardized training groups.

The Chinese version of the RS-14 also demonstrated strong internal consistency reliability in junior nurses (α = 0.939), consistent with prior results in adult Chinese community residents and Hong Kong teenagers using the Chinese version (15, 17).

In terms of structural validity, our study’s CFA findings provided significant support for the RS-14’s two-factor structure, composed of PC and PP, in a sample of Chinese junior nurses. This outcome was consistent with prior research findings, it had a fairer degree than the single factor solution (15, 16, 18). The loadings of all factors were determined to be more than 0.40. This finding suggests that the scale’s two-factor model is stable among nurse populations. Additionally, we utilized the structural validity result as a baseline model for the measurement invariance study. The factor structure of RS-14, on the other hand, differs with culture (13, 15–18). Therefore, when the scale is used in an environment different from the environment in which it was originally developed, reverification is needed.

Before comparing sociodemographic variables, it is necessary to determine a scale’s measurement invariance (19). This measurement is distinct from validity analysis (33). Hence, we assessed the measurement invariance across sociodemographic factors. The 2-factor model of the RS-14 showed invariance for sex, marital status, and status of receiving standardized training but not for academic degrees. The relationship between sociodemographic factors and resilience has not been widely reported, and the conclusions reached are inconsistent. To compensate for the considerable differences in sample sizes between male and female participants (*n* = 516), we randomly selected a subsample of female participants (21, 27). Similarly, random selections were drawn from the other sociodemographic feature groups to match the smaller sample sizes. Although the smallest sample size of a single group was only 179, it was still larger than the minimum sample size required for analysis (24, 34). Since the sample was taken from tertiary hospitals, the education level was relatively high. One possible reason is that to adapt to the sample size of the technical secondary school group, the analysis sample size of education level was relatively small. Due to the small number of people with a technical secondary education in this sample (*n* = 179), in the MGCFA analysis, to avoid the impact of excessive differences in the sample sizes of each group, 179 participants were randomly selected from the groups with professional college and bachelor’s degrees or above to enter the analysis. Although this sample size meets the minimum requirements of CFA (24, 34), previous studies have demonstrated that the sample size impacts the final factor structure (16, 35). CFA was performed on the complete samples of the professional college group (*n* = 5,258) and the bachelor’s degree or higher group (*n* = 1,794), and it was found that the fitting of the 2-factor model improved (not shown in the table). In the associate degree group, the factor loadings ranged from 0.477 to 0.855 (no items below 0.40), and the fitting index was acceptable (RMSEA = 0.095, SRMR = 0.043, TLI = 0.910, CFI = 0.925). Similar results were found in the bachelor’s degree or above group, for which the factor loadings ranged from 0.501 to 0.850 (no items below 0.40), and the fitting index was acceptable (RMSEA = 0.098, SRMR = 0.044, TLI = 0.905, CFI = 0.920). Another possible reason is that there may be other resilience factor structures among people with different academic degrees. Despite this, no previous study has looked at the RS-14’s component structure or measurement invariance across different types of academic degrees. Additionally, the association between educational level and RS-14 has rarely been mentioned in previous studies. The study used the Spearman correlation analysis reported that the overall score on the RS-14 has no meaningful relationship with an academic degree (*r*_*s*_ = –0.06, *P* = 0.33) (16). In a Spanish study using the Brief Resilience Scale to measure resilience, the findings revealed that mental resilience differed significantly across educational levels. (*F* = 3.85; *P* = 0.022), and ANOVA was used to test the difference (36). The measurement invariance of the RS-14 in populations with various educational levels and their mean comparison should be verified in a larger sample. In summary, across a sample of junior nurses with different sociodemographic characteristics, measurement invariance is fundamental in making a reliable comparison of the RS-14 scores aiming to draw a valid statistical inference. For Chinese junior nurses of different genders, marital statuses, and standardized training statuses, the configural invariance indicates that the conceptual framework used to define the two latent factors is the same. In view of the item threshold invariance and the configural invariance model, the RS-14 shows strong factorial invariance concerning sociodemographic variables. To put it another way, it makes sense to compare mean scores between Chinese junior nurses who are male and female, single and married, and who received different standardized training conditions.

Male nurses had a higher latent mean than female nurses. For marital status and status of receiving standardized training, the difference was not statistically significant. We used CFA instead of traditional ANOVA to compare the differences between groups. Although CFA is similar to ANOVA, the former is superior because group comparisons are performed in the context of measurement invariance, while ANOVA assumes only that the given observation score reflects the same level of potential construction in all groups (20). The equal factor loadings and measurement intercept model showed a good fit to the data by gender, marital status, and status of receiving standardized training. For gender, we found a statistically significant difference: the male participants showed a higher latent mean than the female participants, but the difference in actual value was not very large. The findings were confirmed with specific earlier investigations (21). The relationship between marital status, training status, and resilience has not been reported in past studies.

To evaluate scale linkages to other known related factors, we explored relationships between the Chinese version of the RS-14 and other measures in a sample of nurses. The RS-14 scores were strongly and positively connected with the SSRS-USS values. That is, the RS-14 was associated with indicators of social support utilization. However, the RS-14 scores were adversely associated with workplace bullying. This conclusion is consistent with a growing body of research demonstrating resilience’s beneficial associations with other adaptive notions, such as social support (11, 18), and negatively associated with job demands, such as workplace bullying (9).

The internal consistency of the 2-factor model of the RS-14 Chinese version proved that the coefficient alphas were excellent (α = 0.939), indicating high internal consistency among RS-14 scores. Similarly, adequate internal consistency (α = 0.93) between community residents was indicated by the Chinese data. Furthermore, the split-half reliability was 0.897 in the present study. The findings varied from the results published in the literature, and concluded that the RS-14 had good reliability. This finding validates the capacity of the instrument to detect differences in resilience across time. In other studies, test-retest reliability was utilized to uncover differences in resilience across time. We observed that the RS-14 is a reliable and valid instrument for assessing resilience because its reliability and structural validity support its inclusion of a diverse set of protective factors that work cooperatively to create beneficial outcomes in response to stresses (10, 37, 38).

## Strengths and limitations

The current research offers several significant advantages, including the large size of junior nurse groups, measurement invariance testing across sociodemographic features, and sophisticated statistical analyses. This study, however, has several limitations. In our view, the primary limitation was that only junior nurse populations were included, which does not guarantee the representativeness of this population for general nursing evaluations and limits the generalizability of the results. RS-14 is appropriate for adolescents, adults, and the elderly in Chinese communities. Hence, a more age-balanced sample of the RS-14 would enable the assessment of measurement invariance properties.

Furthermore, adversity in an individual’s life may impact resilience, described as an individual’s ability to deal with challenging conditions. Adversity may damage our ability to cope with high stress levels, and adversity can alter resilience over time. However, this study was based on a cross-sectional survey due to the nature of the cross-sectional design, which made it difficult to establish the test-retest reliability and determine whether participants responded consistently to the RS-14 over time, despite its importance for ensuring accurate and replicable measurement. This question needs to be addressed with a prospective investigation in the future. Consequently, investigating the measurement invariance of the RS-14 will require a longitudinal study approach. Given our study’s reliability and validity, future research should explore RS-14 measurement invariance across cultures. Resilience is a personality trait impacted by social, cultural, and environmental factors (39). Due to the cultural differences between the West and the East, the measurement invariance for resilience across cultures should be considered in future research. Despite the limitations reflected in our research, the Chinese version of the RS-14 is an excellent measurement and assessment tool for junior nurses.

## Conclusion

As part of our study, we investigated the psychometric properties of the RS-14 among nurses, concentrating on its measurement invariance across gender, educational level, marital status, and nurses with different standardized training conditions. The RS-14 Chinese version measures psychological resilience in terms of “PC” and “PP.” The findings of our study contributed to our understanding of the factor structure of the RS-14 among Chinese junior nurses. To our knowledge, this is the first study to examine the latent factor structure for the RS-14 among junior nurses in China. Additionally, it is among the few studies to attempt an evaluation of MI across a series of sociodemo-graphic variables among the nurse population. It is confirmed that these two latent factor structures for the RS-14 have sociodemographic-crossed strong factorial MI; hence, it is an effective and reliable tool for measuring the psychological resilience of junior nurses. The correct measurement of psychological resilience levels of junior nurses is expected to provide baseline data that can be used to analyze psychological resilience theory. Therefore, further studies are recommended to improve nurses’ psychological resilience levels through intervention and education programs. A study is needed to establish the resilience of nursing norms in China as well as resilience criteria for the selection of clinical nurses based on the RS-14.

## Data availability statement

The raw data supporting the conclusions of this article will be made available by the authors, without undue reservation.

## Ethics statement

The studies involving human participants were reviewed and approved by The Medical Science Research Ethics Committee in the West China Hospital of Sichuan University approved our study (reference No. 2019-435). The patients/participants provided their written informed consent to participate in this study. Written informed consent was obtained from the individual(s) for the publication of any potentially identifiable images or data included in this article.

## Author contributions

WZ, TS, XW, LP, LZ, and QZ: conception and design. WZ and QZ: administrative support. WZ, TS, XW, and LP: provision of study materials or patients. TS, XW, and LP: collection and assembly of data. WZ and TS: data analysis and interpretation. All authors: manuscript writing and final approval of manuscript.
